# Toxicology Study of Single-walled Carbon Nanotubes and Reduced Graphene Oxide in Human Sperm

**DOI:** 10.1038/srep30270

**Published:** 2016-08-19

**Authors:** Waseem Asghar, Hadi Shafiee, Vanessa Velasco, Vasu R. Sah, Shirui Guo, Rami El Assal, Fatih Inci, Adhithi Rajagopalan, Muntasir Jahangir, Raymond M. Anchan, George L. Mutter, Mihrimah Ozkan, Cengiz S. Ozkan, Utkan Demirci

**Affiliations:** 1 Demirci BAMM Labs, Department of Radiology, Canary Center at Stanford for Cancer Early Detection, School of Medicine, Stanford University, Palo Alto 94304, CA; 2 Department of Computer Engineering & Electrical Engineering and Computer Science, Florida Atlantic University, Boca Raton 33432, FL; 3 Demirci BAMM Labs, Division of Biomedical Engineering, Renal Division, Department of Medicine, Brigham and Women’s Hospital, Harvard Medical School, Cambridge 02139, MA; 4 Mechanical Engineering Department, University of Louisville, Louisville 40292, KY; 5 Department of Electrical Engineering, University of California, Riverside 92521, CA; 6 Center for Infertility and Reproductive Surgery, Department of Obstetrics Gynecology and Reproductive Biology, Brigham and Women’s Hospital, Harvard Medical School, Boston 02115, MA; 7 Department of Pathology, Brigham and Women’s Hospital, Harvard Medical School, Boston 02115, MA; 8 Department of Mechanical Engineering, University of California, Riverside 92521, CA

## Abstract

Carbon-based nanomaterials such as single-walled carbon nanotubes and reduced graphene oxide are currently being evaluated for biomedical applications including *in vivo* drug delivery and tumor imaging. Several reports have studied the toxicity of carbon nanomaterials, but their effects on human male reproduction have not been fully examined. Additionally, it is not clear whether the nanomaterial exposure has any effect on sperm sorting procedures used in clinical settings. Here, we show that the presence of functionalized single walled carbon nanotubes (SWCNT-COOH) and reduced graphene oxide at concentrations of 1–25 μg/mL do not affect sperm viability. However, SWCNT-COOH generate significant reactive superoxide species at a higher concentration (25 μg/mL), while reduced graphene oxide does not initiate reactive species in human sperm. Further, we demonstrate that exposure to these nanomaterials does not hinder the sperm sorting process, and microfluidic sorting systems can select the sperm that show low oxidative stress post-exposure.

Carbon-based nanomaterials (CNMs) are currently under evaluation for various applications in medicine^1^, specifically as delivery vehicles for drugs, proteins, and peptides^2^,^3^,^4^,^5^,^6^, contrast imaging agents^7^,^8^, anti-neoplastic treatment^4^, and in implantable tissues^9^. Despite the numerous advances and potential for new applications, the possible toxic effects of these materials need to be explored to weigh the associated benefits with potential risks. Recent CNM toxicity experiments in animal models demonstrate that injected carbon nanotubes (CNTs)^10^ and graphene oxide (GO)^11^,^12^ migrate through the body and accumulate at different organ sites resulting in damage to cells^13^,^14^ and organs^15^,^16^. Inflammation and formation of small nodules in the lungs^17^,^18^, and induction of atherosclerotic lesions in the artery of animal heart have been reported^16^.

Both GO^19^,^20^ and CNTs^21^ can trigger production of oxidative stress radicals under certain conditions and exposure, leading to cell death and eventual organ injury. These investigations specifically concern the male reproduction, which has been shown to be sensitive to exogenous factors^22^,^23^,^24^, and suffer from ongoing deterioration^25^,^26^. The causes of this deterioration of male reproduction are complex and not well understood, but reactive oxidative stress is believed to be one of the main factors^27^,^28^. In sperm, oxidative stress has been linked to DNA and cell membrane damage resulting in reduced motility^29^. It was shown^10^ that injected multi-walled carbon nanotube (MCNT) suspensions cause reversible testis damage in mice, though, with no measureable adverse effects on mice fertility^10^. Although significant efforts in the past try to understand the toxicity of nanomaterials on various cell types and physiological systems, including reproduction, the effect of CNM on human sperm velocity and oxidative stress generation remain unexplored. Moreover, the effect of nanomaterial exposure on sperm sorting procedures widely used in advanced reproductive technologies (ART) has not been investigated^30^,^31^. Therefore, it is very important to investigate whether the sperm exposed to toxic materials can be sorted with current sperm sorting methods including microfluidic based sperm sorting^30^,^31^,^32^.

SWCNTs, graphene, and their functional forms are representative CNMs, which are frequently investigated for their toxicological effects on different organs and cells^10^,^33^,^34^. Carbon nanotubes and graphene are intrinsically not soluble in water, therefore, these materials are generally modified by chemical functionalization to enhance their solubility and potential to attach various drug molecules^35^,^36^. Functionalized materials are generally more miscible in water, hence, mostly used for cell toxicity studies. Therefore, for this study, we chose functionalized carbon based material (SWCNT and RGO) as representative materials. Here, we evaluate the *in vitro* influence of carboxylate-functionalized single-walled carbon nanotubes (SWCNT-COOH) and reduced graphene oxide (RGO) on human sperm at various concentrations (1–25 μg/mL) and incubation times (0.5–3 hours). To understand the material-sperm interactions, we analyzed sperm viability, velocity, and generation of superoxide and nitric oxide stresses as a function of nanoparticle (NP) concentration and incubation period (Fig. 1a–j). Further, we investigated the feasibility of microfluidic-based sperm sorting method for isolating healthy sperm from semen samples exposed to nanomaterials.

## Results

### Particle Size Distribution

To demonstrate the aggregation and size distribution of SWCNT-COOH and RGO in HTF-HEPES + 1% BSA buffer over a period of time (0 min, 30 min, and 3 hour), particle size was analyzed (Supporting Figure S1). In the case of SWCNT-COOH, we observed that nanotubes showed good dispersion at t = 0 minute with nanoparticle diameters of less than 10 nm. We also observed few nanotubes aggregates with larger sizes (~1 μm) in the samples (Supporting Figure S1a, t = 0 plots). At 30 minute and 3 hour time-points, we observed particles aggregation with sizes of 1–5 μm, especially in the case of higher nanotube concentrations (25 μg/mL). We observed similar trend in the case of RGO suspensions, where 25 μg/mL RGO suspensions had higher aggregation with effective particle sizes of 1–5 μm (Supporting Figure S1b).

### Sperm viability and kinematics

**Figure 2:**
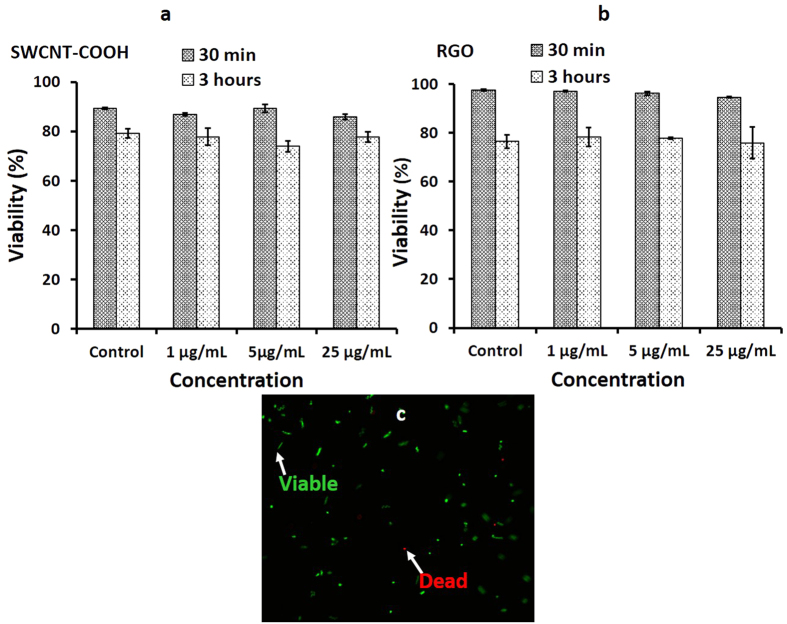
Sperm viability analysis after incubation with nanomaterials. Viable sperm population (control, 1, 5, and 25 μg/mL) after incubating with **(a)** SWCNT-COOH and **(b)** RGO for 30 minutes and 3 hours. **(c)** Fluorescent image of viable (green) and dead (red) sperm were used for viability quantification.

**Figure 3:**
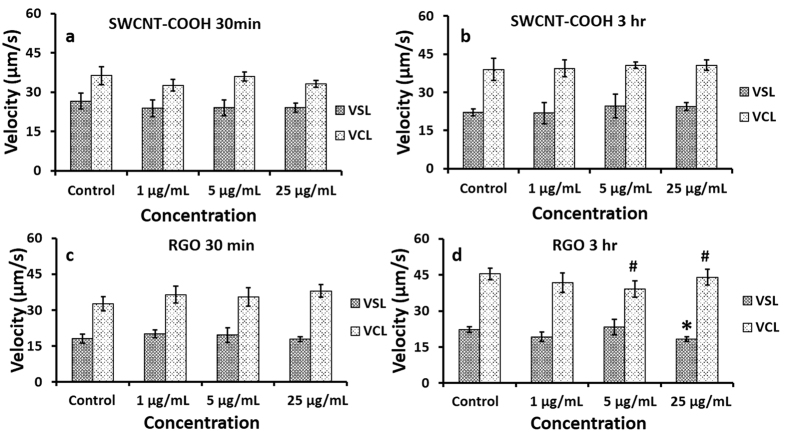
Sperm kinematic analysis at various SWCNT-COOH/RGO concentration and exposure time. Linear (VSL) and curvilinear (VCL) sperm velocity for sperm samples (control, 1, 5, and 25 μg/mL) incubated with SWCNT-COOH for **(a)** 30 min and **(b)** 3 hours, as well as, incubated with RGO for **(c)** 30 minutes and **(d)** 3 hours. *p value < 0.05 between VSL of sperm incubated with RGO and control samples, N = 3. ^**#**^p value < 0.05 between VCL of sperm incubated with RGO and control samples, N = 3. Error bars represent a standard error of the mean.

Viability of human sperm was quantified after a 30 minutes or 3 hour incubation period with SWCNT-COOH or RGO at various concentrations (Fig. 2a–c). We observed no significant changes in sperm viability due to SWCNT-COOH or RGO exposure (*p value* > 0.05). We also analyzed the sperm for kinematics; straight linear velocity (VSL) and curvilinear velocity (VCL). We observed no significant changes in VCL and VSL of sperm incubated with SWCNT-COOH at different time points; 30 minutes and 3 hours (*p value* > 0.05) (Fig. 3a,b). This indicated that the sperm exposure to SWCNT-COOH did not alter the sperm kinematic performance. In the case of RGO exposure, we observed no significant differences in sperm velocity after 30 minutes of incubation (*p value* > 0.05) (Fig. 3c). On the other hand, sperm velocity decreased after RGO exposure at higher concentrations (5, 25 μg/mL) and longer incubation time (3 hours) (Fig. 3d).

### Reactive oxygen species (ROS) generation

**Figure 4:**
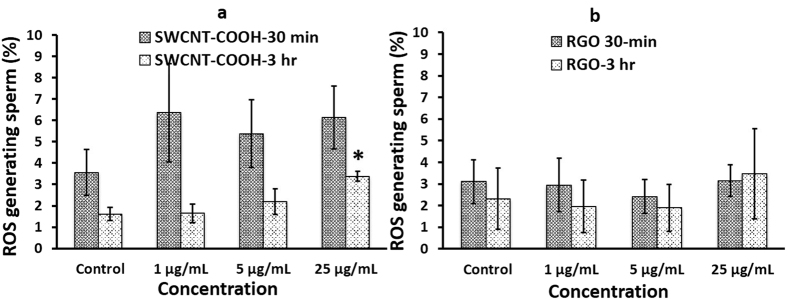
Superoxide generation at various SWCNT-COOH/RGO concentration and exposure time. Sperm population generating reactive oxide stress (ROS) for sperm samples incubated with **(a)** SWCNT-COOH and **(b)** RGO at NP concentrations of 1, 5, and 25 μg/mL for two incubation periods of 30 minutes and 3 hours. *p value < 0.05 between sample treated with SWCNT-COOH and control, N = 3. Error bars represent a standard error of the mean.

Nanoparticle cytotoxicity investigations have attributed oxidative stress as a catalyst to pulmonary inflammatory pathology^37^. Superoxide ROS generated by sperm were measured and quantified after incubation with either SWCNT-COOH or RGO (Fig. 4a,b). RGO exposure did not produce significant ROS compared to control sperm samples (*p value* > 0.05) (Fig. 4b). In the case of SWCNT-COOH, we observed significant superoxide ROS generation after nanoparticle exposure at a higher concentration (25 μg/mL) and longer incubation time (3 hours) (*p value* < 0.05) (Fig. 4a).

### Nitric oxide generation

**Figure 5:**
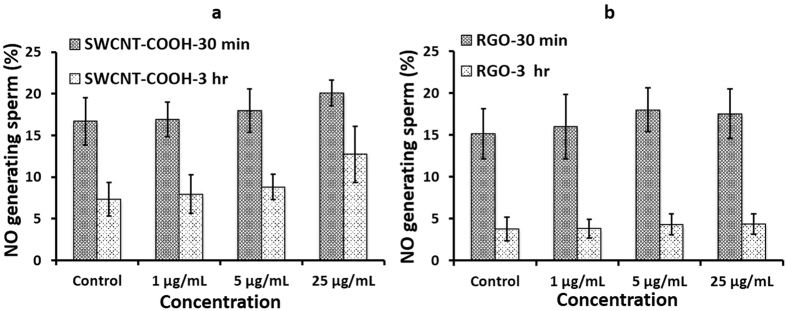
Nitric oxide species generation due to SWCNT-COOH/RGO concentration and exposure time. Sperm population generating nitric oxide species (NOS) for sperm samples incubated with **(a)** SWCNT-COOH and **(b)** RGO at concentrations of 1, 5, and 25 μg/mL for two incubation periods of 30 minutes and 3 hours.

Nitric oxide (NO) reactive species is another marker for sperm damage, often linked to impaired sperm movement^38^. We did not observe any significant NO production in sperm after incubation with SWCNT-COOH or RGO at various concentrations (1–25 μg/mL) and time points (0.5–3 hours) (*p value* > 0.05) (Fig. 5a,b). These results suggest that RGO did not induce substantial NO to be able to trigger apoptosis during the time frames and concentrations specified in this study.

### Multivariate evaluation of nanotoxicologic effect

In the experiments, by changing incubation time and concentration of either SWCNT-COOH or RGO, we evaluated their nanotoxicologic effects in terms of viability, VCL, VSL, ROS, and NOS levels of sperm. To better understand their effect, we first classified these assays into two groups: (i) viability assays and (ii) functionality assays (*i.e.*, VCL, VSL, ROS, and NOS). As demonstrated in Fig. 2, the individual groups were evaluated using an ANOVA test, and we did not observe any statistical significant change in sperm viability when we applied SWCNT-COOH and RGO to sperm in different incubation time and concentrations (*p value > 0.05*). (ii) To evaluate the effect of SWCNT-COOH and RGO exposure over sperm functionality (VCL, VSL, ROS, and NOS), we used a multivariate analysis (MANOVA), where we designed three different statistical assessments (p value is set to 0.05): (a) As the first analysis, we compared the multivariate effect of SWCNT-COOH and RGO with their repeated measurements including various concentrations (1, 5, and 25 μg/mL) and different time domains (30 min and 3 hour). Here, independent parameters were defined as SWCNT-COOH and RGO, whereas dependent parameters were determined as VCL, VSL, RGO and NOS levels. As a result, we observed a significant multivariate effect between SWCNT-COOH and RGO experiments, F (44, 82) = 1.748, p = 0.015; Wilks’ Lambda = 0.08010. (b) As an internal comparison, we also evaluated the multivariate effect of SWCNT-COOH and its controls in different time domains (30 min and 3 hour) and various concentrations (1, 5, and 25 μg/mL) with repeated measurements. In the second analysis, independent parameters were determined as SWCNT-COOH and control, whereas dependent parameters were stated as VCL, VSL, RGO and NOS levels. There was no multivariate effect observed in SWCNT-COOH experiments when we compared the results with control samples, F (28, 48) = 1.705, p = 0.051; Wilks’ Lambda = 0.08388. (c) Similarly, we employed another internal comparison for RGO and its controls in different time domains (30 min and 3 hour) and concentrations (1, 5, and 25 μg/mL) using a multivariate analysis with repeated measurements. In this analysis, independent parameters were defined as RGO and its controls, whereas dependent parameters were determined as VCL, VSL, RGO and NOS levels. Here, we did not observe any multivariate difference between RGO and the control groups, F (28, 48) = 1.432, p = 0.135; Wilks’ Lambda = 0.11307. According to MANOVA tests with repeated measurements, we had two outcomes: (i) SWCNT-COOH and RGO affected differently over sperm functionality, (ii) whereas these did not have any significant change in sperm functionality when we compared them with their controls at different time points and various concentrations.

### Sorting sperm post-nanomaterial exposure

**Figure 6:**
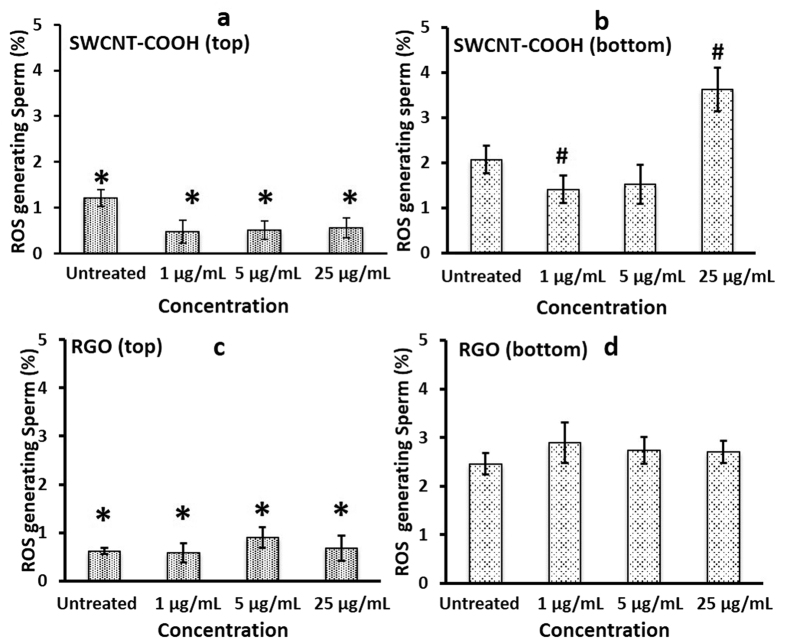
ROS generation sperm population after sorting. Sperm population generating reactive oxide species (ROS) for **(a)** sorted sperm collected from top chamber (n = 6, Modified Z-Scores method was used to detect outliers in the data^69^. One data point out of 6 readings has Z-Score > 3.5, therefore it is neglected) and **(b)** sperm collected from bottom chamber of microfluidic device after samples are incubated with SWCNT-COOH (n = 6). Sperm population generating reactive oxide species (ROS) for **(c)** sorted sperm collected from top chamber (n = 6) and **(d)** sperm collected from bottom chamber of microfluidic device after samples are incubated with RGO (n = 6). *p value < 0.05 between sperm collected from top and bottom of the filter. ^**#**^p value < 0.05 between sperm samples treated with SWCNT-COOH and untreated sample.

In nature, the sperm are tested for their fitness in the vaginal tract, which sorts out the sperm that is most likely to create a successful zygote and pregnancy. Today, during *in vitro* fertilization procedures, we lack tools to sort sperm that are exposed to potential nanotoxic effects. To addresses this challenge, we tested our recently developed microfluidic device to sort sperm. We previously reported^31^ that sperm sorted using microfluidic device showed significantly lower ROS generation compared to whole semen suspensions^31^. In this study, we have used this microfluidic device to sort sperm samples treated with SWCNT-COOH or RGO. We tried to mimic vaginal tract channels in a microfluidic-chip system^31^ by using a micron sized porous membrane and evaluated its potential utility in selecting the sperm that are the most motile and the least affected post-exposure to nanoparticles (Fig. 6a–d). To evaluate our hypothesis, we incubated sperm populations with SWCNT-COOH and RGO for two and half hours. We injected the NP treated sperm samples and allowed sperm to randomly swim up for 30 minutes. Sorted sperm were recovered from the top compartment and measured for the ROS generating sperm population (Fig. 6a–d). Sorted sperm showed a significant decrease in ROS generating population by ~1.3 fold for untreated sample, ~3 fold for SWCNT-COOH at 1 and 5 μg/mL and by ~6 fold for SWCNT-COOH at 25 μg/mL (p value < 0.05) (Fig. 6a,b). For RGO-sperm groups, the chip sorted sperm also showed a significant decrease in ROS production by ~4 fold for untreated, 1 and 25 μg/mL samples and by ~3 fold for 5 μg/mL sample (p value < 0.05) (Fig. 6c,d).

## Discussion

Toxicity of CNMs including SWCNT-COOH and RGO depends on various factors, such as dosage, time of exposure, cell types, and aggregation degree. For example, in the literature, carbon nanotubes are reported to have no significant toxic effects on peritoneal macrophage^39^, clonal pheochromocytoma cells^40^, HeLa cells, and Jurkat cells even at concentration higher than used in this study (25 μg/mL)^41^. On the other hand, multiple studies reported that carbon nanotubes showed toxic effects for Human MSTO-211H cells^42^, A549 human pneumocytes^43^, Human lung epithelial H460 cells^33^, and HeLa cell lines^41^. These reports demonstrate that CNMs may or may not have toxicological effect on various cell types based on their dosage, surface functionalization, and exposure time. The effects of carbon based materials on human sperm *in vitro* have not been thoroughly investigated. A study by Bai *et al*. reported an *in vivo* evaluation of mouse sperm after repeated administration of various doses of carbon nanotubes^10^. They reported that the nanomaterial exposure had no significant effect on sperm viability, which was consistent with our viability results for the *in vitro* toxicity evaluation on human sperm.

In previous *in vivo* studies, dosages of CNTs administered intravenously to mice range between a single dosage of 5 mg/kg or five dosages of 5 mg/kg administered every 3 days, where nanoparticle solution concentration was fixed to 1 mg/mL^10^. They reported that after a single dosage of 5 mg/kg of CNT-COOH, approximately 151 ng of nanotubes were found in every gram of testes tissue 24 hour after injection administration. Bai *et al*. also reported an additional dosage scheme consisting of 5 mg/kg dosages administered every 3 days. However, due to the short lifetime of radiolabels used, CNT accumulation could not be analyzed. Bai *et al*. expected a higher accumulation of CNTs in the testes with this higher dosage scheme. This was due to the increasing trend of nanotubes in the testes found within 24 hours of administering a single dosage. However, during *in vivo* studies, the exact amount of nanomaterials at which sperm were exposed was unknown. In the case of previous *in vitro* carbon nanotube toxicology studies on various different cell types such as lung cells, HeLa cells, macrophages, typical CNT doses tested ranged 0.25–1000 μg/mL^44^,^45^,^46^. Based on these previous *in vitro* and *in vivo* reports, we have selected the CNM concentrations in the lower range (1–25 μg/mL of sperm sample for this study) of previously reported concentrations.

Sperm motility parameters are used as indicators of potential reproductive abnormalities. Sperm mobility parameters have been known to decline as a result of exposure to environmental^47^,^48^, industrial^49^,^50^, and pharmaceutical agents^51^. In such cases, VSL and VCL were reported to decrease. In our experiments, we observed no significant change in sperm motility when sperm were incubated with SWCNT-COOH (p value > 0.05). Whereas, we observed a significant decrease in sperm motility when sperm were incubated for longer period (3 hours) with RGO (p value < 0.05). This decrease in sperm velocity after RGO exposure may be the result of physical trapping of sperm by RGO sheets (Supporting Figure S7), in agreement with the previous findings, where bacteria were trapped within aggregating reduced graphene sheets in a suspension^52^.

Oxidative stress has been demonstrated to boost cellular damage due to the production of ROS, which are highly reactive free radicals known to have adverse effects on sperm function and morphology. Studies have shown that oxidative stress is one of the main causes of male infertility, showing decreased sperm motility and viability, increased midpiece morphology defects, and lipid peroxidation of sperm membranes^53^. We observed no significant superoxide ROS or NO generation after nanoparticle exposure under the experimental conditions except when sperm were exposed to higher concentrations (25 μg/mL) and longer incubation time (3 hours) (Fig. 4a). These findings suggest potential toxic effects on sperm when exposed to SWCNT-COOH (Fig. 4a) under these conditions. The size and functional groups of carbon-based nanomaterials play important role in determining their toxic effects on sperm^41^. RGO particles are less miscible in HTF than SWCNT-COOH and are larger in size (Supporting Figures S1 and S2), hence, may not easily penetrate cell membrane as reported previously^54^. Functionalization of SWCNT with –COOH makes them more miscible in HTF and they can be uptaken by cell membrane using endocytosis, phagocytosis^41^, or nanopenetration^39^, which leads to more ROS generation compared to RGO.

It is important to note that the population of sperm generating both superoxide ROS and NO was more significant at the 30 minute time point and decreased by the third hour (Figs 4 and 5). This behavior can be indicative of species generated early on by capacitation. The generation of ROS is one of the initial occurrences among the many series of events that take place in sperm capacitation^55^. Our results are consistent with previous studies, which showed that superoxide production began soon after the sperm were incubated in media. This superoxide production peaked within the first 20 minutes and slowly decreased after incubation for 2 hour and 45 minutes^56^,^57^. Further, this behavior can be indicative of an initial burst of oxygen species generated by the initial centrifugation that sperm cells were subjected for swim-up isolation. Measurement of superoxide in sperm isolated by swim-up had a higher and more sustained peak after 20 minutes of incubation with media, and this oxidative stress subsequently decreased^57^.

We have also employed advanced microfluidic sorting techniques to sort sperm after NP exposure showing that NP exposure does not affect the selection of motile sperm during the procedure. Conventional sperm sorting methods such as swim-up and density gradient based methods may not be as efficient as microfluidics in selecting healthy sperm from sperm population exposed to toxic nanomaterials. These alternate techniques require centrifugation, which have been shown previously to damage sperm and generate higher ROS^31^,^58^,^59^,^60^. The microfluidic technique demonstrated here is a passive technique, where semen is injected in the lower compartment and the random motion of the sperm enables sperm separation without centrifugation. As various sperm sorting methods are currently being used during IVF procedures, it is necessary to investigate their efficiency to select healthy sperm for reduced ROS levels.

## Conclusion

This study demonstrated the first pilot toxicity evaluation of functionalized SWCNTs and RGO on human sperm. Sperm exposure to nanomaterials did not result in significant changes in sperm viability. However, there were some significant changes in sperm velocity and oxidative stress due to reactive oxygen species. The generation of oxidative stress raises concerns because it is possible that these materials may have adverse effects on male fertility at higher quantities over a longer period. We also concluded that nanoparticle exposure to sperm does not appear to hinder the sperm sorting procedures using an advanced microfluidic method. These results encourage further exploration studies to analyze the effects of exposed sperm on fertilization and zygote development.

## Methods

### Preparation of Graphene Oxide

Graphene Oxide (GO) was first prepared with modified Hummer’s method as previously described^61^,^62^,^63^. Then GO was filtrated and rinsed several times with deionized (DI) water. 32 mg GO was dispersed in 4mL PBS (phosphate bufferd saline, PBS is mixture of NaCl (137 mmol/L), KCl (2.7 mmol/L), Na_2_HPO_4_ (10 mmol/L), and KH_2_PO_4_ (1.8 mmol/L) buffer, followed by 15 minutes of mild sonication (at 150 W power) to prepare 8mg/mL GO stock sample. SEM and AFM images of synthesized GO are shown in Supporting Figure S2.

### Reduction of Graphene Oxide to Reduced Graphene Oxide

GO powder was dried at 50 °C in a vacuum oven. GO was reduce to reduced graphene oxide (RGO) by exposing it to hydrazine for two hours at room temperature. Hydrazine is the most common and effective reducing agent used to convert GO to reduced graphene oxide RGO via hydrothermal treatment^64^. The dispersion was sonicated for 30 min (150 W), followed by centrifugation at 1000 rpm/min for 20 min to remove large particles in the solution. Fourier transform infrared spectroscopy (FTIR) spectrum of GO and RGO confirmed the reduction of GO (Supporting Figure S3).

### Preparation of SWCNT-COOH

Introduction of –COOH on to the end of SWCNT was conducted according to literature^65^. Briefly, SWCNTs suspended in nitric acid (HNO_3_) solution (2M) were sonicated for 30 minutes to obtain homogeneous dispersion of SWCNTs in the acid medium. Oxidation process was carried out by refluxing the mixture at 130 °C for 16 hours. The use of nitric acid reflux oxidized the SWCNTs mildly and introduced carboxylic groups at defect sites (primarily at the tips), while the graphitic sidewalls were chemically inert. Purification and isolation of CNTs from the oxidation mixture was performed carefully before subjecting them to the conjugation process. Filtration of the reaction mixture was carried out with Millipore polycarbonate filter paper with a pore size of 0.2 mm, followed by repeated washings with DI water before suspending the CNTs in DI water. To obtain a pure sample, multiple rinses with DI water were conducted with centrifugation to remove any acid residue. Rinse was not stopped until the pH of top water layer reached 6. The sample was dispersed in water and used for sperm experiments. Raman spectrum of SWCNT showed lower D peak and G’ peak that indicated the high quality of SWCNT was synthesized (Supporting Figure S4). FTIR spectrum showed the functionalization of SWCNTs with –COOH (Supporting Figure S5).

### Carbon Nanomaterial Suspension Preparation

RGO and SWCNT-COOH were suspended in PBS, pH 7.4 (Gibco Invitrogen, # 10010-023) at 3 different concentrations of 1, 5, and 25 mg/mL. PBS contains Potassium Phosphate monobasic (KH_2_PO_4_) 144 mg/L, Sodium Chloride (NaCl) 9000 mg/L, Sodium Phosphate dibasic (Na_2_HPO_4_-7H_2_O) 795 mg/L. Carbon nanomaterial solutions were sonicated (40 kHz, Fisher Scientific, # 15-335-60) for 1 hour at room temperature prior to being mixed with sperm samples.

### Characterization of Particle Size and Distribution of SWCNT-COOH and RGO

The particle size and distribution of SWCNT-COOH and RGO in HTF-HEPES + 1% BSA medium were evaluated by using Dynamic Light Scattering (DLS) technique (Zetasizer Nano ZS, Malvern Instruments, UK) as previously reported^54^. For this experiment, SWCNT-COOH and RGO nanoparticle suspensions were prepared in HTF medium to concentrations of 1, 5 and 25 μg/mL. The solutions were analyzed at three time points 0, 30 min, and 3 hours after preparation.

### Human Sperm Preparation

Human semen vials (1.0 cc each, Intracervical Insemination specimen vials) were purchased from California CryoBank following IRB 2012P000590 regulations and stored in liquid nitrogen. HTF-HEPES (InVitroCare, Frederick, MD) was supplemented with BSA (Sigma, St. Louis, MO) and used for sperm preparation and sorting. Sperm samples were thawed at 37 °C in the water bath for 15 minutes before use.

### Concentration Analysis

Using a Makler counting chamber, a 1 μL sperm sample was manually analyzed for concentration according to manufactures’ instructions.

### Swim-Up Method

Each 1mL semen vial was divided into two fractions of 475 μL in centrifuge tubes. 4 mL of HTF-HEPES 1% BSA medium was added over the semen and centrifuged at 400 g for 10 minutes. The supernatant was discarded very carefully without disturbing the pellet. Following this, 500 μL of media was added from sidewalls meticulously while avoiding disruption of the pellet. These tubes are placed in the incubator, inclined at an angle around 45° and incubated at 37 °C for 45 minutes. By tilting the tubes at 45°, we increase the surface between the medium and the semen, improving the capability of the sperms to swim out and reach the medium. The supernatant (motile sperm) of each tube is gently removed from each vial, leaving the pellet behind. Swim-up sperm cell population is recorded using a Makler counting chamber and used for kinematic, reactive oxygen and nitric oxide toxicity studies.

### Motility Analysis

Sperm motility was assessed as described in WHO laboratory manual for sperm analysis (“WHO laboratory manual for the Examination and processing of human semen,” 2010)^66^. Specifically, motile sperm were collected after performing the swim-up method. Motile cell suspensions (~2 million sperm/mL) were mixed with appropriate volumes of SWCNT-COOH and RGO suspensions so that nanoparticle concentration in the sample was 1, 5, or 25 μg/mL. Sperm-carbon nanomaterial samples were placed in the incubator at 37 °C for 0 min, 30 min, and 3 hr. After each time point, approximately 6 μL of sample was placed on a 18 mm × 18 mm coverslip to form a liquid channel of 20 μm in depth as suggested by WHO manual^66^. Motile sperm were imaged using brightfield light microscopy (Carl Zeiss) with a 20x objective under ambient conditions as previously reported^30^,^31^,^67^. Videos were recorded for 5 seconds at three different locations of the coverslip. Videos were converted to jpeg images and kinematic parameters, straight linear velocity (VSL) and curvilinear velocity (VCL) were determined using a sperm tracking MATLAB code. At least 200 motile spermatozoa were tracked and analyzed for each sample.

### Viability Analysis

A LIVE/DEAD sperm viability kit (L-7011, Molecular Probes) was used for analyzing sperm samples. Propidium iodide (PI) was used to stain dead sperm and SYBR 14 Dye was used to stain live cells, according to the manufacturer’s protocol. SYBR 14 dye was added to the sperm samples for a final concentration of 100 nM followed by an incubation for 5 minutes at 37 °C. For the staining of dead sperm, PI dye was added to the samples to a final concentration of 10 μM and incubated for 5 more minutes. Fluorescence microscope was used to image the smeared sperm samples on a glass cover slip using green and red emission filters. At least 200 sperm were counted for each sample.

### Reactive Oxygen Species (ROS) Detection

Flow cytometry was used to assess the generation of ROS produced by the nanomaterials using two different fluorescent dyes, dihydroethidium (DHE) and SYTOX green. DHE reacts with superoxide anion producing two flourochromes binding to the sperm DNA and imparting red fluorescence. On the other hand, Sytox Green, an indicator for cell viability, produces green fluorescence when cells are dead. All compensation control samples were first prepared by adding 20 μL of hydrogen peroxide to 200 μL of sperm suspension, followed by incubation at 37 °C for 1 hour. Then, the compensation control samples were divided into four groups and treated with dyes as follows. For the negative control, no dye was added. For the second sample, DHE at 5 μM was added. For third sample, SYTOX green at 50 nM was added. For the fourth sample, both DHE and SYTOX at 5 μM and 50 nM, respectively, were added. Finally, the samples were incubated at room temperature for 20 minutes, followed by flow cytometric analysis.

For experimental samples, swim-up sperm suspensions were respectively mixed with Single Walled Carbon Nanotube–COOH and Reduced Graphene Oxide suspensions at three concentrations (1 μg/mL, 5 μg/mL or 25 μg/mL). These samples were then placed in a cell culture incubator at 37 °C for 30 minutes or 3 hours. Samples were then incubated with DHE (5 μM) and Sytox (50 nM) for 20 minutes at room temperature, followed by flow cytometric analysis (Becton Dickinson) for measuring ROS at each time point. Experiments were repeated six times (N = 6). Emission measurements were done using 530/30 band pass (green) and 585/42 band pass (red) filters for FL1 and FL2, respectively coupled with Argon laser excitation at 488 nm. At-least 10000 sperm cells were examined and the non-sperm events were gated out.

### Nitric Oxide Species (NOS) Detection

The generation of NOS by nano-materials was assessed using a fluorescent dye DAF-2-DA (4,5-diaminofluorescein-2/diacetate). L-NAME (NG-nitro-L-arginine methyl ester) (in DMSO) an analogue of L-arginine was used as a competitive inhibitor of NOS while Sodium Nitroprosside (SNP) was used as a NO donor. All compensation control samples were first prepared by adding 10 μM of DAF-2-DA dye to 200 μL sperm suspensions. Then, the compensation control samples were divided into four groups and treated with dyes as follows. For the negative control, no dye was added. For second sample, DAF-2-DA dye was added. For the third sample L-NAME at 3 mM with DAF-2-DA dye was added. For the fourth sample, SNP at 400 μM with DAF-2-DA dye was added. All control samples were incubated for 2 hours at 37 °C followed by dye incubation for 30 minutes at room temperature.

For experimental samples, swim-up sperm suspensions were respectively mixed with Single Walled Carbon Nanotube–COOH and Reduced Graphene Oxide suspensions at three concentrations (1 μg/mL, 5 μg/mL or 25 μg/mL). These samples were then placed in a cell culture incubator at 37 °C for 30 minutes and 3 hours. Samples were then incubated with DAF-2-DA (10 μM) for 30 minutes at room temperature, followed by flow cytometric analysis (Becton Dickinson) for measuring NOS at each time point. Experiments were repeated three times (N = 3). Fluorescence was measured using excitation wavelength of 488 nm and emission wavelength of 530 nm using FL1. At-least 10000 sperm cells were examined and the non-sperm events were gated out.

### Filter device fabrication and experimentation

#### Chip Assembly

The PMMA (3 mm thick; McMaster Carr, Atlanta, GA) and DSA (120 μm thick, St. Paul, MN) were cut using a laser cutter (Versa Laser, Scottsdale, AZ). The design for the chip was generated on Coral Draw4 and implemented onto USLE Engrave software for cutting. Primary components of the MSS included one 3 mm PMMA cut to an area of 50mm × 30 mm (bottom chamber) and another cut to an area of 30 mm × 30 mm (top chamber). A 0.6-mm injection point was also cut into the bottom PMMA sheet at a 5-mm distance from the chambers. Cylinders of 20 mm diameter were cut into both PMMA components. The bottom PMMA chamber was first attached to glass slide using DSA. Top PMMA chamber was aligned and attached with bottom chamber using DSA. The Nuclepore track-etched polycarbonate membrane filter (Whatman Ltd, 25 mm diameter, 8 μm pore size) were sandwiched between two PMMA chambers during chip assembly^31^ (Supporting Figure S6). The components were sterilized by washing thoroughly with 70% ethanol followed by air-drying before assembling inside the cell culture hood.

#### Experimentation

The swim-up sperm suspensions were respectively mixed with SWCNT–COOH and RGO suspensions at three concentrations (1 μg/mL, 5 μg/mL or 25 μg/mL) and incubated at 37 ^o^C for 2 hours and 30 minutes. Following the incubation, the filtered sperms from the top of the chip were carefully collected, without tampering the polycarbonate membrane. The unfiltered or leftover sperm suspension inside the chip was then extracted by creating a small incision on the membrane filter. Both sperm suspensions were subject to flow cytometric analysis for measurement of ROS and NOS following the procedures as described above. Experiments were repeated six times (N = 6).

#### Statistical analysis

Each experiment was repeated at least three times, and the results of sperm parameters were expressed as mean ± SEM (standard error of the mean). To understand the statistical changes in the experimental groups, we performed two types of statistical analyses: (i) one-way analysis of variance (ANOVA), which we compared the individual groups, and (ii) multivariate ANOVA (MANOVA), which we evaluated two independents (SWCNT and RGO) and dependent parameters (*e.g.*, VCL, VSL, ROS, and NOS) with repeated measurements. In MANOVA analysis, we employed Wilks’ Lambda according to equal distribution of sample size as indicated in the literature^68^. MANOVA statistical analyses were performed using Minitab (Release 17, Minitab Inc., State College, PA). A value of p < 0.05 was considered statistically significant.

## Additional Information

**How to cite this article**: Asghar, W. *et al*. Toxicology Study of Single-walled Carbon Nanotubes and Reduced Graphene Oxide in Human Sperm. *Sci. Rep*. **6**, 30270; doi: 10.1038/srep30270 (2016).

## Supplementary Material

Supplementary Information

## Figures and Tables

**Figure 1:**
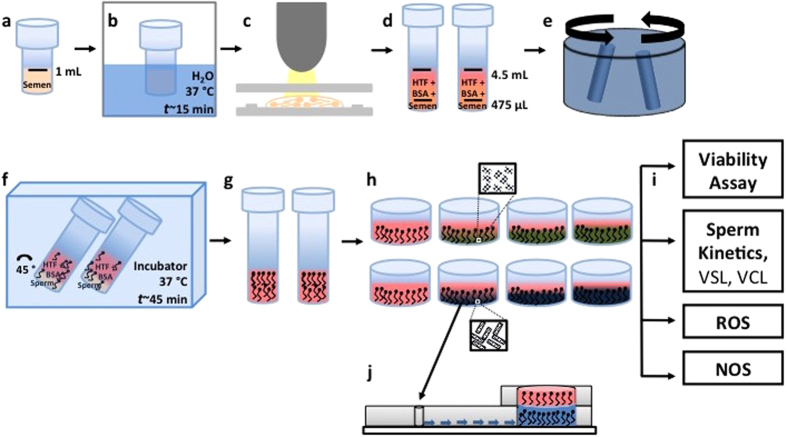
SWCNT-COOH/RGO concentration and time dependent sperm toxicity study. **(a)** 1 mL semen vials were obtained from a cryobank. **(b)** Semen sample was thawed in a water bath at 37 °C for 15 min. **(c)** Sperm concentration and motility were quantified using a Makler hemocytometer. **(d)** Semen samples were mixed with HTF and 1% BSA and **(e)** centrifuged. **(f)** Sperm samples were placed in an incubator and motile sperm were allowed to swim up to the top media layer. **(g)** Supernatant containing motile sperm was collected and distributed in 8 wells**. (h)** Sorted semen were incubated with SWCNT-COOH and RGO at various concentrations; 1, 5, 25 μg/mL, and for 30 minutes or 3 hour time points. **(i)** Viability, sperm kinetic parameters, ROS, and NO were measured. **(j)** Sperm incubated with nanomaterials were processed using a microfluidic device to investigate the effects of nanomaterials on sperm sorting process.
