# Supplementary material for: Toxicology Study of Single-walled Carbon Nanotubes and Reduced Graphene Oxide in Human Sperm
Source: Sci Rep. 2016 Aug 19;6:30270. doi: 10.1038/srep30270 (PMC4990966; doi:10.1038/srep30270)
Supplement: Supplementary Information [file srep30270-s1.pdf]

Supporting Information

SUBJECT AREAS: NANOTOXICOLOGY, CARBON NANOMATERIALS

Toxicology Study of Single-walled Carbon Nanotubes and Reduced Graphene Oxide in Human Sperm

Waseem Asghar[1,2], Hadi Shafiee[3], Vanessa Velasco[3,4], Vasu R. Sah[3], Shirui Guo[5], Rami El Assal[1], Fatih Inci[1], Adhithi Rajagopalan[3], Muntasir Jahangir[3], Raymond M. Anchan[6], George L. Mutter[7], Mihrimah Ozkan[5], Cengiz Ozkan[8], and Utkan Demirci[1,3]*

[1] Demirci BAMM Labs, Department of Radiology, Canary Center at Stanford for Cancer Early Detection, Stanford School of Medicine, Stanford University, Palo Alto, CA 94304

[2] Department of Computer Engineering & Electrical Engineering and Computer Science, Florida Atlantic University, Boca Raton, FL 33432

[3] Demirci BAMM Labs, Division of Biomedical Engineering, Renal Division, Department of Medicine, Brigham and Women's Hospital, Harvard Medical School, Cambridge, MA 02139

[4] Mechanical Engineering Department, University of Louisville, Louisville, KY 40292

[5] Department of Electrical Engineering, University of California, Riverside, CA 92521

[6] Center for Infertility and Reproductive Surgery, Department of Obstetrics Gynecology and Reproductive Biology, Brigham and Women's Hospital, Harvard Medical School, Boston, MA 02115

[7] Department of Pathology, Brigham and Women's Hospital, Harvard Medical School, Boston, MA 02115

[8] Department of Mechanical Engineering, University of California, Riverside, CA 92521

* Corresponding Author E-mail: utkan@stanford.edu

(a) Size distribution of SWCNT-COOH in HTF media

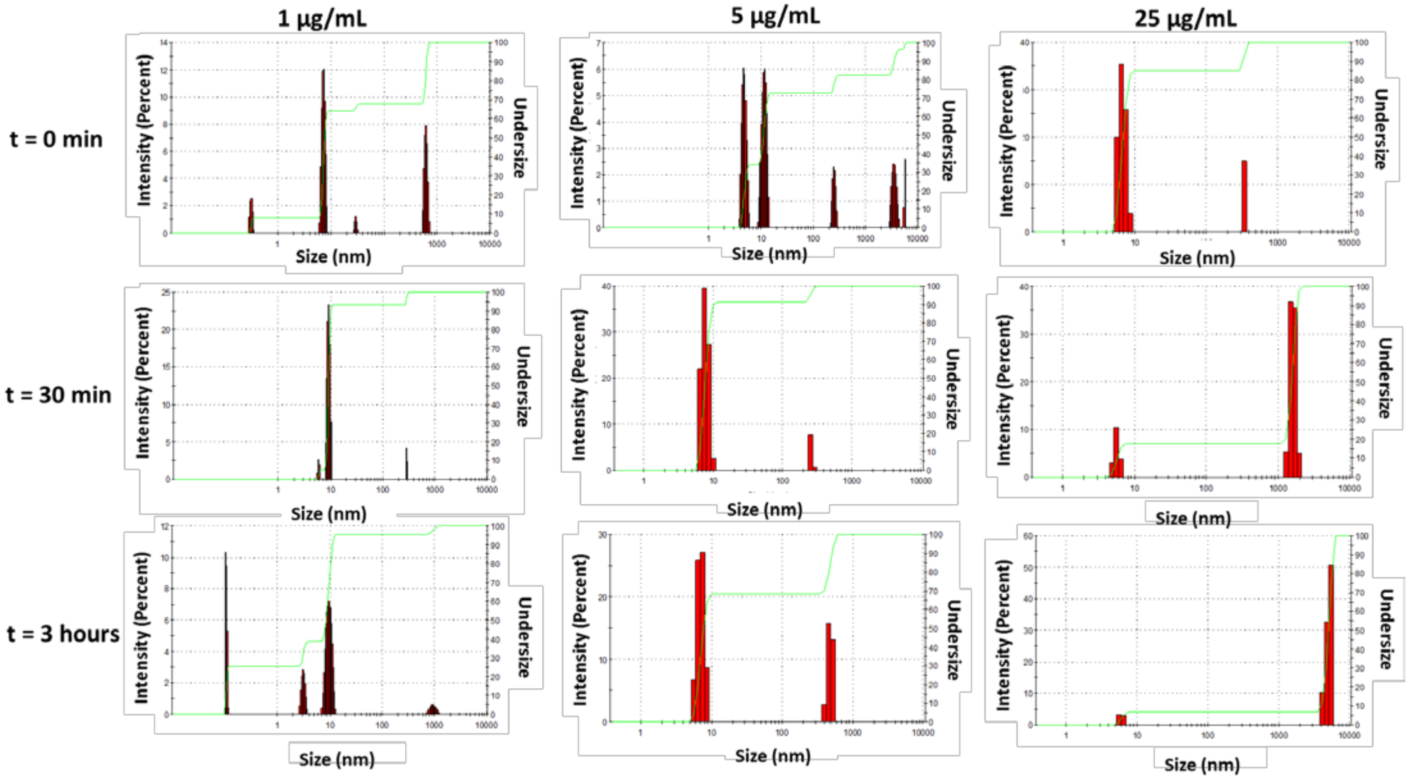

(b) Size distribution of RGO in HTF media

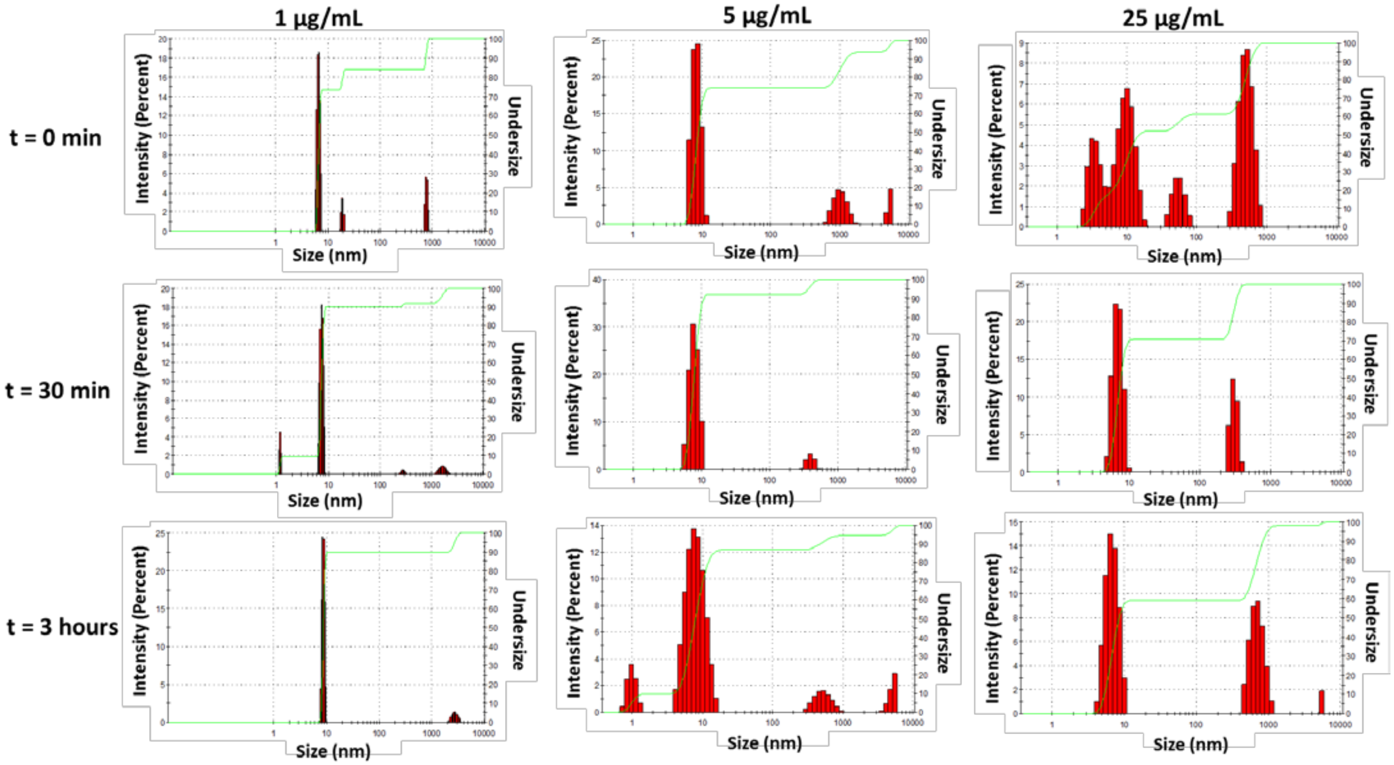

Supporting Figure S1. Size distribution plots for (a) SWCNT-COOH and (b) RGO in HTF buffer at three different concentrations (1µg/mL, 5µg/mL, and 25µg/mL) and time points (0min, 30min, and 3hours) using a dynamic light scattering (DLS) technique (Zetasizer Nano ZS, Malvern Instruments, UK).

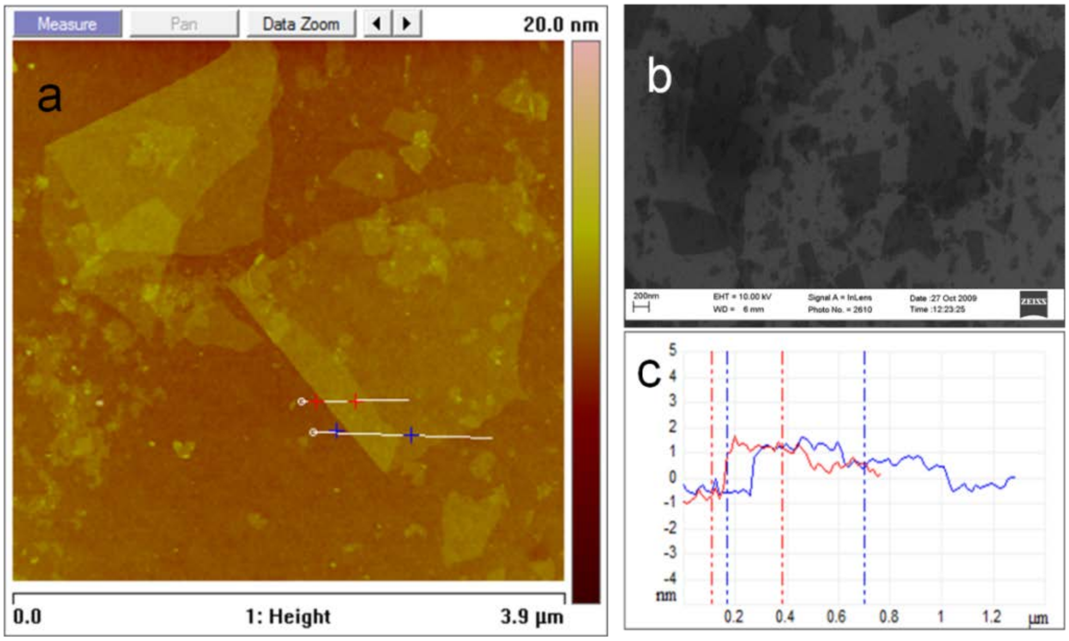

Supporting Figure S2. AFM and SEM images of as-synthesized graphene oxide (GO) single layer: **a,** AFM image shows that single layer GO was successfully prepared, **b,** SEM image shows different sizes of GO, Scale bar = 200nm, **c,** height profile of AFM image.

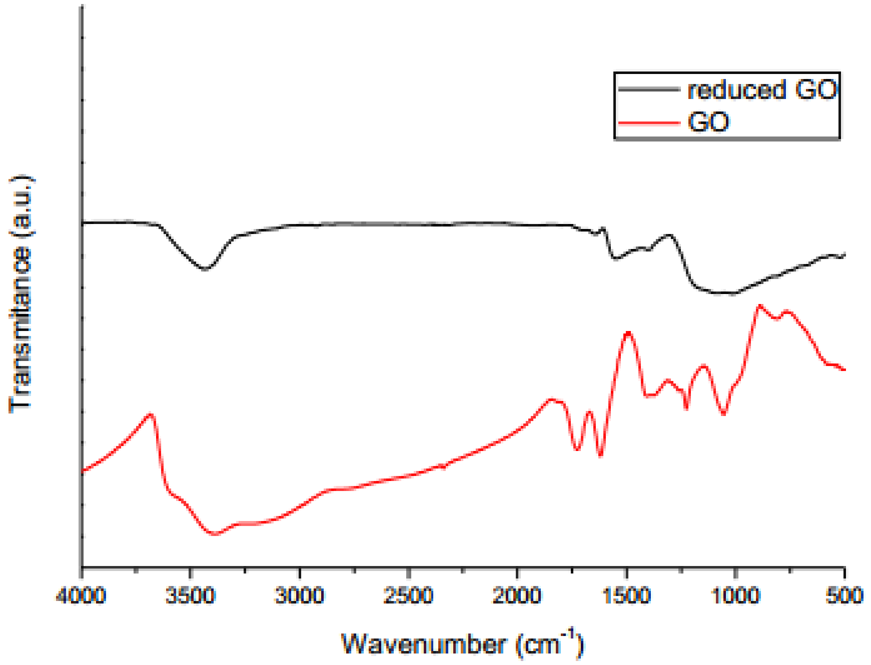

Supporting Figure S3. Fourier Transform Infrared (FTIR) Spectra of GO and reduced GO.

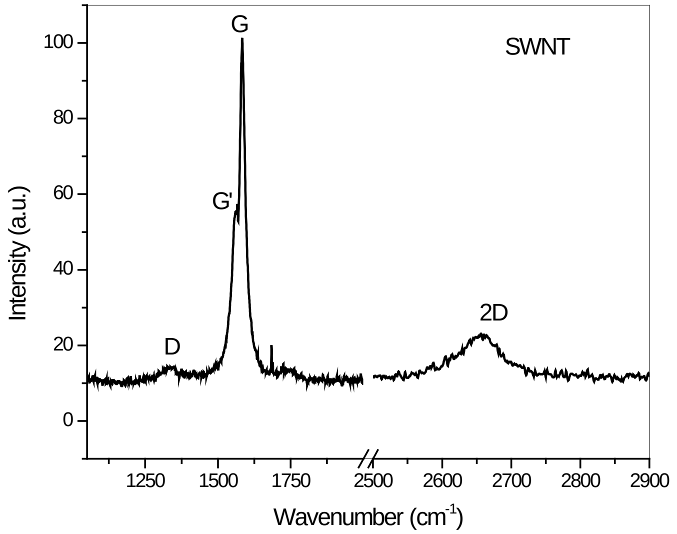

Supporting Figure S4. Raman spectra of untreated SWCNT. The lower D peak and G’ peak indicate the high quality of the tube.

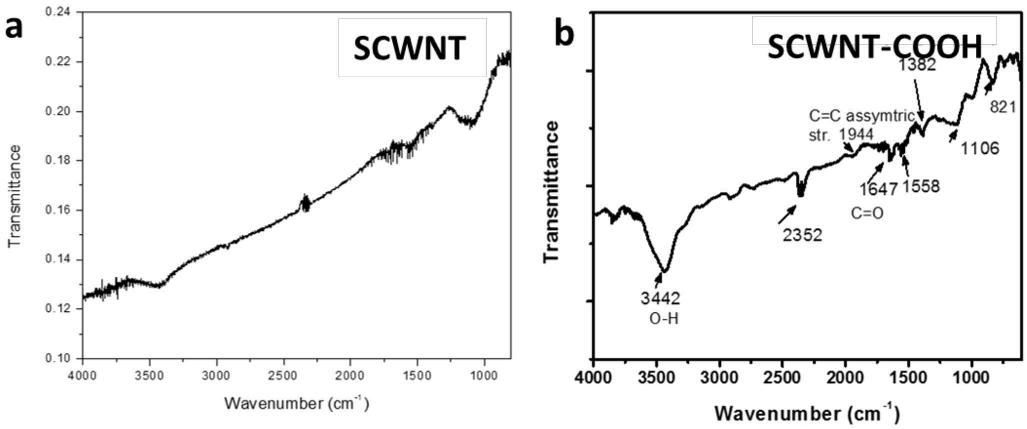

Supporting Figure S5. Fourier Transform Infrared (FTIR) Spectra of (a) SWCNT and (b) -COOH functionalized SWCNT. The appearance of O-H group and C=O group in FTIR spectra shows the functionalization of SWCNTs with –COOH.

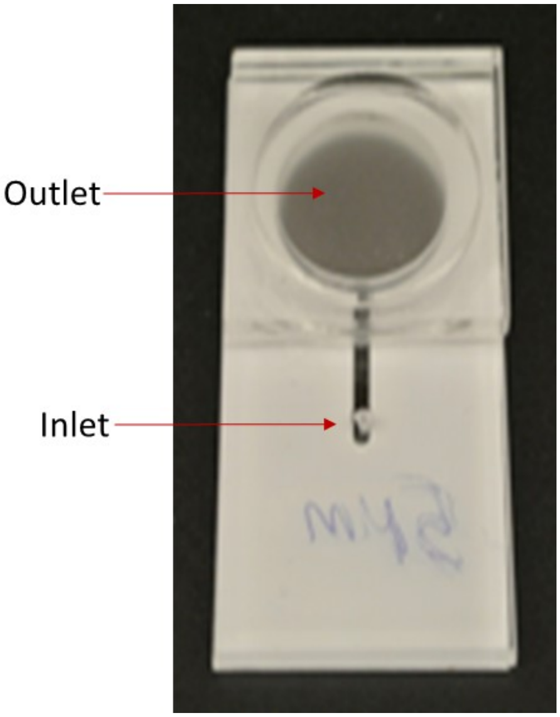

Supporting Figure S6. Image of the sperm sorting microfluidic device showing the inlet and outlet for sperm injection and collection. The device enables sorting healthy sperm from a sample population.

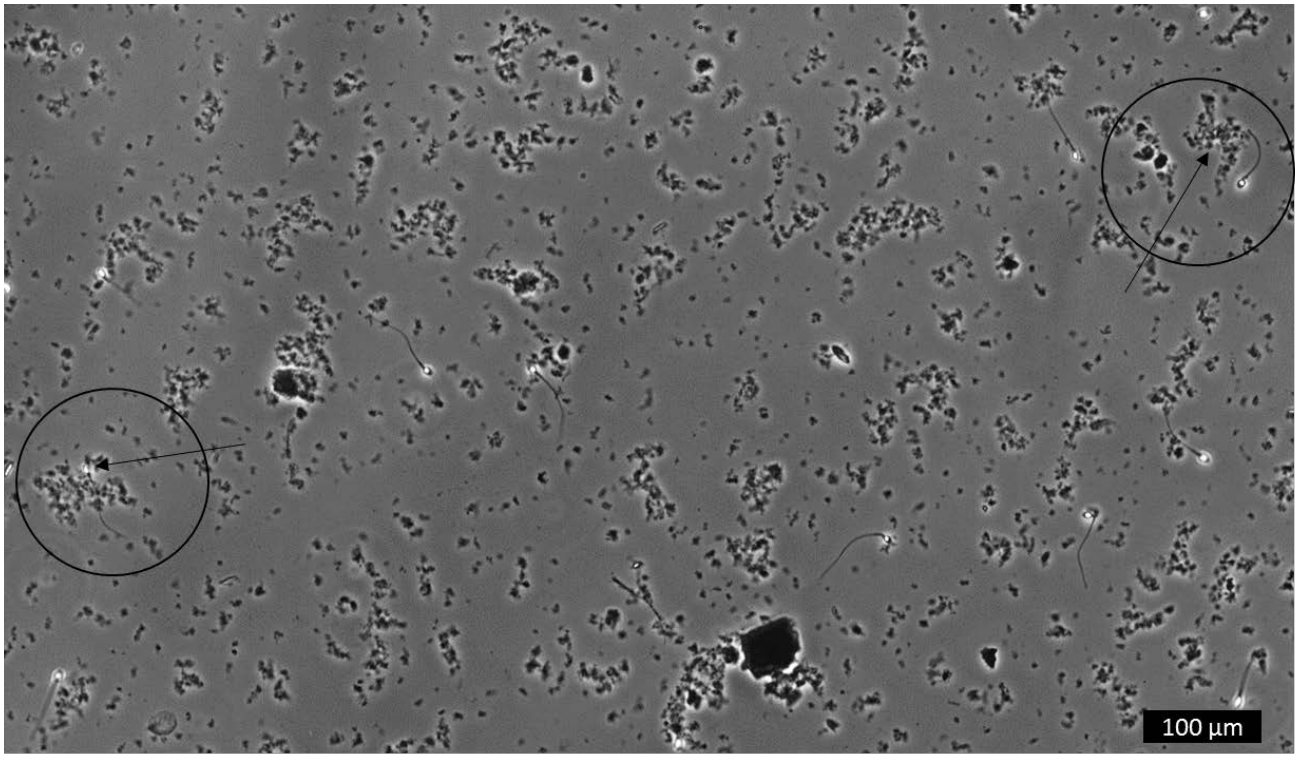

Supporting Figure S7. Bright-field image of sperm exposed to RGO after 3 hour incubation. Circles show some areas in the image where sperm are trapped with RGO. Arrows points to the head of trapped sperm.
